# Breastfeeding environment and experiences at the workplace among health workers in the Upper East Region of Ghana

**DOI:** 10.1186/s13006-023-00565-1

**Published:** 2023-06-14

**Authors:** Clement Kubreziga Kubuga, Juliana Tindana

**Affiliations:** 1 Nutritional Sciences Department, University for Development Studies, Tamale, Ghana; 2 Ghana Health Service, Upper East Region, Bolgatanga, Ghana

**Keywords:** Breastfeeding, Workplace breastfeeding environment, Health worker, Ghana

## Abstract

**Background:**

Employed mothers have lower rates of breastfeeding, including health workers who are supposed to be advocates for breastfeeding. These working mothers need a supportive workplace environment to breastfeed, yet Ghana’s breastfeeding policy neither mentions the workplace breastfeeding environment nor offers any information on it.

**Methods:**

A convergent parallel mixed-methods design was used in this study to determine: facilities with a complete breastfeeding support environment (BFSE); breastfeeding challenges experienced; coping strategies and motivators for breastfeeding among health workers in the Upper East Region of Ghana; and Management’s awareness of the need for an institutional breastfeeding support policy. Quantitative and qualitative data were analyzed using descriptive statistics and thematic analysis respectively. The research was conducted from January to April 2020.

**Results:**

All facilities (39) had incomplete BFSE and management representatives of health facilities (39) did not have and were not aware that their respective facilities needed to have a specific workplace breastfeeding policy that fed into the national policy agenda. Breastfeeding challenges at workplaces included: lack of private space for breastfeeding; inadequate support from co-workers and management; emotional stress; and inadequate breastfeeding breaks and work options. Women adapted to these challenges by employing coping strategies such as: bringing children to work with / without caretakers; leaving children at home; seeking support from co-workers and family members; feeding children with supplementary foods; adding annual leave to maternity leave; breastfeeding in cars / offices; and sending children to daycare. Interestingly, the women were still motivated to breastfeed. Health benefits of breastmilk, the convenience and readily available nature of breastmilk, moral obligation to breastfeed, and cheap cost of breastmilk emerged as key motivators to breastfeed.

**Conclusion:**

Our findings suggest that health workers have poor BFSE and are faced with numerous breastfeeding challenges. There is a need for programs that improve BFSE in health facilities.

## Background

Breastfeeding can be affected by maternal employment and workplace policies [1]. Some studies have indicated that workplace breastfeeding support such as provision of adequate time for expressing milk, family-friendly policies, flexible schedules, part-time work options, access to childcare, and access to suitable facilities to breastfeed or express milk promote breastfeeding practices [2–4]. Additionally, work-related issues have been identified as a major reason as to why mothers do not initiate breastfeeding or stop breastfeeding sooner [1, 5]. As such, public policies are required for working mothers to effectively enforce their choice to optimally breastfeed [1].

Currently in Ghana, there is a policy measure to promote early initiation, exclusive breastfeeding and complementary feeding [6] with lots of technical challenges on implementation. Interestingly, the policy neither mentions the workplace breastfeeding support environment (BFSE) nor offers any information on it. A facility is deemed to have a fully functional BFSE if it has the following features: a private area for breastfeeding and milk expression; flexible break and / or work options for nursing mothers; back-to-work breastfeeding education for other health professionals / coworkers; and a lactation support policy that is clear and consistent. Ghana adapted a comprehensive Infant and Young Child Feeding strategy in 2007 [7] via the UNICEF / World Health Organization (WHO) Global Strategy for Infant and Young Child Feeding initiative, which might have contributed to the observed reduction in prelacteal feeding from 18% to 2008 to 15% in 2014 [8, 9] and an increase in the proportion of babies breastfed within the first hour of birth from 52% to 2008 to 56% in 2014 [8]. Despite these gains, only 43% [10] of infants younger than 6 months in 2017 were exclusively breastfed compared to 52% in 2014 and 63% in 2008 [8]. The proportions are even lower among professional workers (10.3%) [11] which includes health workers who are supposed to be advocates for breastfeeding. Despite the fact that studies on health professionals’ breastfeeding practices in our study context are essentially non-existent, a recent study found that just 62.5% of health professionals (nurses) practiced exclusive breastfeeding (EBF) [12]. This EBF rate is far below the the global target of 70% [13].

The median duration of EBF has also declined progressively over the years for the Ghanaian population; a decline from 4.4 months (2008) to 2.5 months in 2014. There has also been a drastic decline in the EBF rates after 2–3 months when the duration of paid maternity leave ends.

Considering the undesirably low breastfeeding rates, a relatively short maternity leave couple with a breastfeeding policy that lacks specifics on the BFSE, this study aims to: (1) find the proportion of health facilities with a complete workplace BFSE; (2) document challenges / barriers and coping strategies of health care providers (women) in health facilities without a complete workplace BFSE; (3) document the motivators for breastfeeding among health care providers (women) in health facilities without a complete workplace BFSE; and (4) find out managements’ awareness of the workplace breastfeeding support policy.

As health workers are supposed to be advocates for breastfeeding, the primary focus of this research is on health workers and the health care system in relation to breastfeeding. Additionally, to the best of the authors’ knowledge, this is the first study to particularly look at the workplace BFSE, challenges, motivators, and coping strategies of women in health facilities in the research setting.

## Methods

**Fig. 1:**
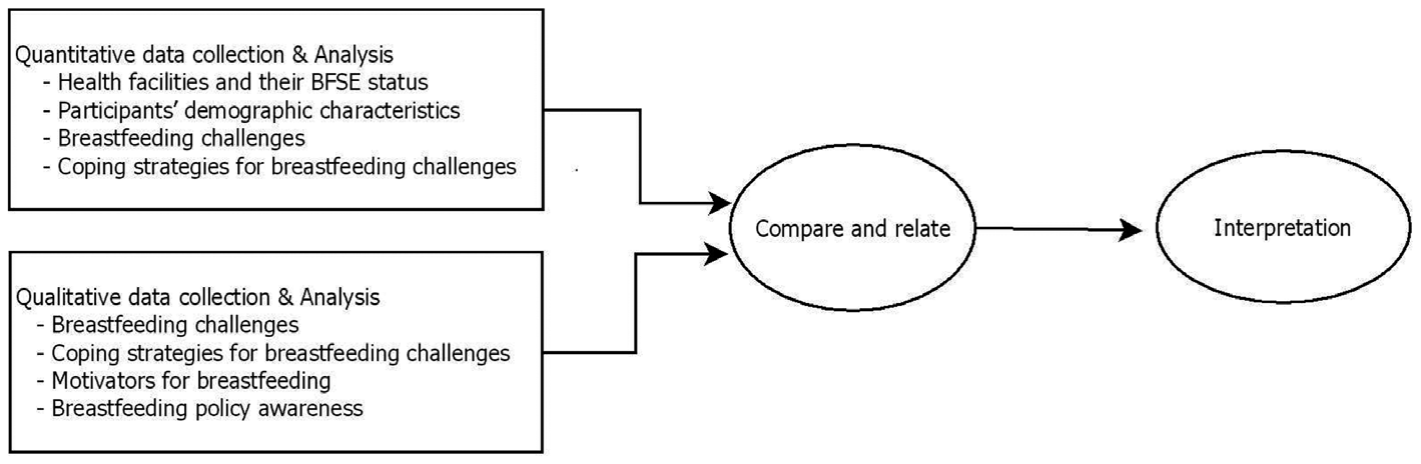
Research process

To gain deeper understanding of the topic, a convergent parallel mixed-methods design was used in this study. Quantitative and qualitative components were conducted simultaneously during the same phase of the research process. In the research process (Fig. 1), two datasets were collected, analysed separately, and compared. The research was carried out from January to April 2020 in the Upper East Region of Ghana, one of the regions with the highest malnutrition prevalence in Ghana.

### Quantitative data collection

The study covered health facilities: health directorates, hospitals, health centres, and Community-based Health Planning and Services (CHPS) compounds. Participants were mainly female nurses who had been in their current health facilities for not less than six months. They should have breastfed or are currently breastfeeding while engaged by their health facility. Participants also included management representatives who met the aforementioned criteria. Participants were recruited through convenience sampling as they were expected to reflect a complete experience of breastfeeding at the workplace. Participants’ information on socio-demographic characteristics, breastfeeding challenges, and coping strategies was obtained using a semi-structured questionnaire. A 4-point checklist was used to check BFSE status of participants’ respective facilities.

### Qualitative data collection

**In-depth interviews** were used to obtain information on health facility management’s awareness level of workplace breastfeeding support policy, and the need for workplace breastfeeding support policies among management representatives.

**Focus group discussions** (three) were used to explore breastfeeding challenges / barriers, coping strategies, and motivators at the workplace. Convenience sampling was used to draw participants from health facilities for focus group discussions. To ensure trustworthiness and data quality, researchers kept an audit trail. Additionally, researchers took notes on discussions as well as nonverbal cues such as facial and body expressions. Focus group discussions were facilitated by the authors. All sessions were tape recorded. After each focus group, the researchers shared notes to check the consistency of the information captured. Each focus group was made up of eight participants and conducted with the aid of a guide / prompts (*E.g.: What are / were the barriers for breastfeeding at your current workplace? In spite of the challenges, what motivated you to continue to breastfeed?*) to help obtain nuanced information. It is worth noting that both quantitative and qualitative data on challenges of breastfeeding and coping strategies were collected for the purpose of the validation of our findings.

### Statistical analysis

#### Quantitative data

The estimated sample size (n = 157) was computed using N = t^2^ x p(1-p) / m^2^, the normal standard deviation (t) at a confidence level of 95%, margin of error (m) of 5%, proportion (p) of EBF among professional workers of 10.3% [11] and non-response rate of 10%. Data analyses were carried out using SAS 9.4 (SAS Institute Inc., Cary, NC, USA.).

Descriptive statistics were used to present variables (sociodemographic characteristics, breastfeeding challenges, coping strategies, and BFSE status of health facilities). Missing data were not included in computing descriptive statistics. A facility was said to have a complete BFSE when it had: private space for breastfeeding and milk expression; flexible breaks and / or work options for breastfeeding mothers; back to work breastfeeding education for other health professional / co-workers; and a lactation support policy for clarity and consistency [14]. BFSE was further classified as moderate and poor if a facility had two / three and one / none of the above stated provisions respectively. At each facility, information on the aforementioned criteria was sought via questioning and direct inspection, assisted by the use of a predesigned 4-point checklist. A score of four meant a facility had a complete BFSE.

#### Qualitative data

Focus group discussions were transcribed and analysed with the aid of Excel spread sheets using colour coding. We related specific codes from the first phase into broader subcategories. Selective coding was used to integrate all of the relationships between categories and subcategories into overarching themes. Coding for interviews was done in the same manner as the focus group discussions.

## Results

### Socio-demographic characteristics of respondents

The results in Table 1 indicate that the majority of the participants were either between 20 and 30 years old (52%) or 31– 40 years old (46%). Nearly all the participants (92%) were married and live with their husbands (83%). Most of the participants were Christians (74%) and Frafras (36%). Almost all the participants (96%) had a college / university / polytechnic education and work in: health centers (38.2%), CHPS compounds (24%), regional hospitals (17%), and district hospitals (15%). A significant proportion of the participants did not belong to any women’s group (58%), lived within a nuclear family (66%), and lived in urban areas (61%). The majority of the participants’ spouses were employed (95%), were civil servants / public sector workers (78%), and had a college / university / polytechnic education (92%).

**Table 1:** Sociodemographic variables of health workers in the Upper East region of Ghana (*n* = 157)

Names of variables		*n*	%	Mean ± SD
Age group of respondents				30.70 **±** 3.94
	20–30 years	82	52	
	31–40 years	73	47	
	41–50 years	2	1	
Marital status of respondents				
	Married	145	92	
	Singled / never married	8	5	
	Separated / divorced	2	1	
	Widowed	2	1	
Respondent currently living with husband				
	Yes	121	83	
	No	24	17	
Religion of respondents				
	Islam	41	26	
	Christianity	116	74	
Family type system				
	Nuclear family	104	66	
	Extended family	53	34	
Ethnicity of respondents				
	Frafras	57	36	
	Bulisa	13	8	
	Kusase	13	8	
	Nankana/Kasana	19	12	
	Akans	14	9	
	Waali / Dagaati	8	5	
	Dagomba	7	4	
	Others	26	17	
Respondents’ Education level				
	No formal education	1	1	
	Informal education	1	1	
	Basic education	2	1	
	Senior education	2	1	
	College / university / polytechnic	151	96	
Respondents’ Spouse’s education level				
	No formal education	1	1	
	Informal education	1	1	
	Basic education	3	2	
	Senior education	6	4	
	College / university / polytechnic	134	92	
Respondents’ Places of work				
	District Health Directorate	10	6	
	Regional Hospital	26	17	
	District Hospital	23	15	
	Other Hospital	1	1	
	Health Centers	60	38	
	CHPS Compound	37	24	
Respondent’s spouse’s working status				
	Yes	138	95	
	No	7	5	
Respondents’ spouse’s occupation				
	Farmers	6	4	
	Civil / public servants	107	78	
	Traders / own business	16	12	
	Artisans	1	1	
	Others	8	6	
Belonging to women’s groups				
	Yes	66	42	
	No	91	58	
Residence of respondents				
	Rural	62	39	
	Urban	95	61	

NOTE: Due to running off decimal places, percentages may not sum up to 100%. Missing values were not included in data presentation.

Abbreviations: *SD* standard deviation

Table 2 provides information on breastfeeding and the prevailing breastfeeding environment in health facilities in the Upper East Region of Ghana. The study covered 39 health facilities, including four health directorates (10%), seven hospitals (18%), fifteen health centres (39%), and thirteen CHPS compounds (33%).

**Table 2:** Female health workers’ breastfeeding experience and environment in the Upper East region of Ghana

Variable		*n* (%)
**Health facilities** (***n*** = **39**)		
Facility level or type	Regional / District / Municipal Health Directorate	4 (10)
	Regional / District / other Hospitals	7 (18)
	Health centers	15 (38)
	CHPS compounds	13 (33)
**Breastfeeding support environment requirements in facilities**		
Flexible work breaks / options	Present	39 (100)
	Absent	0 (0)
Education for co-workers	Present	0 (0)
	Absent	39 (100)
Room for breastfeeding	Present	1 (3)
	Absent	38 (97)
Lactation policy	Present	0 (0)
	Absent	39 (100)
**Workplace breastfeeding support environment in facilities**		
Environment classification	Poor	38 (97)
	Moderate	1 (3)
	Complete	0 (0)
**Breastfeeding and workplace (*n*** **= 157)**		
Breastfeeding / breastfed at workplace	Yes	122 (78)
	No	35 (22)
Average number of times a participant is / was able to breastfeed at the workplace per day	Four or more times	110 (90)
	Two to three times	8 (7)
	Once	3 (2)
	Not allowed to bring a child to the workplace	1 (1)
Environment type mother breastfed in	Poor	116 (95)
	Moderate	6 (5)
	Complete	0 (0)

NOTE: Due to running off decimal places, percentages may not sum up to 100%. Missing values were not included in data presentation

On BFSE requirements, facilities had: flexible work breaks / options (100%), breastfeeding room (3%), education for co-workers (0%), and lactation support policy (0%). None of the facilities had complete BFSE (0%), 3% and 97% of the facilities had moderate and poor BFSE respectively.

78% of the participants either breastfed or are breastfeeding at the workplace and breastfed / breastfeeds in; *poor BFSE (95%)* and *moderate BFSE (5%).* Among the mothers who breastfed at the workplace, 90% of them breastfed / breastfeed their infants four or more times (breastfeeding on demand), two to three times (7%), and once (2%) during working hours. Some mothers (1%) were not allowed to bring children to work.

Table 3 presents both quantitative and qualitative results of breastfeeding challenges and the respective coping strategies participants adapted.

**Table 3:** Workplace breastfeeding challenges and adaptations from a semi-structured questionnaire and focus groups discussions

Structured questionnaire (*n* = 157) n (%)		n (%)	Focal groups discussion (*n* = 18)
			**Key Themes**:
**Breastfeeding challenges**			***Workplace challenges***
	Difficulty in combining work and breastfeeding	65 (41)	Difficulty in combining work and breastfeeding
	No private space for breastfeeding	53 (34)	No private space for breastfeeding
	Emotional stress	10 (6)	
	Others	9 (6)	
	Inadequate breastfeeding breaks	8 (5)	Inadequate breastfeeding breaks
	No support from co-workers and management	6 (4)	No support from co-workers and management
	Inadequate work options	6 (4)	***Psychological challenges***
			Emotional stress
			**Key Themes**:
			***Workplace adaptation strategies***
**Coping strategies**	Bring child to work with caretaker	26 (26)	Bring child to work with caretaker
	Bring child to work without a caretaker	24 (24)	Bring child to work without a caretaker
	Leave child at home	18 (18)	Leave child at home
	Support from co-workers	17 (17)	Support from co-workers
	Breastfeed in the car / office	7 (7)	Breastfeed in the car / office
	Support from family members	5 (5)	Add annual leave to maternity leave
	Send baby to daycare	3 (3)	***Family and social support strategies***
			Support from family members
			Send baby to daycare
			Alternative feeding approaches

NOTE: Due to running off decimal places, percentages may not sum up to 100%. Missing values were not included in data presentation

On breastfeeding challenges (quantitative data), participants reported difficulty in combining work and breastfeeding (41%), lack of private space for breastfeeding (34%), emotional stress (6%), inadequate breastfeeding breaks (5%), others (6%), inadequate work options (4%), and no support from co-workers and management (4%) as their main challenges. To cope with the stated challenges, the participants; bring their children to work with a caretaker (26%), bring their children to work without a caretaker (24%), leave their children at home when coming to work (18%), get support from their co-workers (17%), breastfeed in their cars or offices (7%), get support from their family members (5%), and send babies to daycare (3%).

For the qualitative data, key breastfeeding challenges and coping strategies identified were the same as those in the quantitative results as indicated below:

### Workplace challenges

Key workplace challenges were largely obstacles that hindered breastfeeding; mothers had no designated place to breastfeed so they had to breastfeed in cars / offices (Lack of private space for breastfeeding), mothers had specified period in which they had to work and could not work per schedules that suits them so they had to seek external support (family or caretaker) or come to work with babies at their backs (inadequate work options), mothers had complains coming from some co-workers and management due to their breastfeeding status interfering with work (no support from co-workers and management), mothers were not also able to breastfeed on demand or as they wished due to short and limited breastfeeding breaks (inadequate breastfeeding breaks).

The underlisted gives some details of mothers’ expressions of their challenges:

- Lack of private space for breastfeeding.
  “For me I don’t have anybody to help me, I take care of the child during working hours, so what I do is, I have to take a break from my work and breastfeed her in the office.” *(FD2, N1)*.
  “At the workplace when you are breastfeeding you see that people will be entering and you will not have much time to breastfeed so I think if you are in the house, it is better.” *(FD2, N6)*.
- Inadequate work options.
  “If you are at the workplace you don’t always have much time yet, there are no alternatives, it will be interfering with the work”. *(FD1, N4)*
  “The workside there is limited time, you may want to breastfeed and they say come and do this, you may have to leave your child and go and attend to the person before you come back.” *(FD3, N8)*.
- No support from co-workers and management.
  “Because you are having a baby, you will be exempted from doing some work which makes some of your colleagues to be complaining and you end up having issues with some of them”. *(FD1, N5)*
- Inadequate breastfeeding breaks.
  “I also want to add that breastfeeding should be on demand, with our limited break time it is very difficult to feed on demand, that is why it is [more] difficult to feed at work than at home.” *(FD2, N8)*.
  “If you are at the workplace you don’t always have much time, it will be interfering with the work.” *(FD3, N1)*.

### Psychological challenges

Mothers were mostly emotionally stressed in combining tight work schedules and breastfeeding (psychological challenges). The under-listed are examples of how some mothers expressed their emotional stress experience.

- Emotional stress.
  “I was emotionally stressed because baby was constantly disrupting work”. *(FD1, N8)*
  “There is emotional stress involved in breastfeeding and working. You want the work and the child, you just have to adjust”. *(FD2, N6)*

### Workplace adaptation strategies

Mothers adapted some behaviours that they used to adjust to the challenges at their workplaces (coping strategies). With regards to the stated challenges (lack of private space for breastfeeding, inadequate breastfeeding breaks, inadequate work options, no support from co-workers and management, psychological challenges), some mothers breastfed in cars / offices; others identified friendly colleagues and asked for support; some carried their babies at their backs during working hours while others combined their annual and maternity leaves to enable them to exclusively breastfeed their children up to six months before they returned to work (workplace adaptation strategies). Some mothers also sought the support of family members or caretakers to enable them to combine work and breastfeeding, and others sent their kids to daycare centres (family and social support strategies).

The underlisted gives some details of mothers’ expressions of their coping strategies:

- Leave children at home when coming to work.
  “I had somebody in the house who could take care of my child, so I just express and then leave it behind so when I get [a] chance around 12 o’ clock, I asked for permission and go to breastfeed my child.” *(FD3, N6)*.
- Bring children to work with a caretaker.
  “Some of us because of the work, we have to get a caretaker who can follow you to the work-side, so that as you are working, the person will be taking care of your child, so that when your child needs something she calls you or small time you now go and see your child.” *(FD2, N8)*.
- Bring children to work without a caretaker.
  “For my situation, it wasn’t easy because with my baby, my baby will not even go to anybody, so you come to work, the baby is at your back, you are working, you have to break, let the baby suck and sometimes, you don’t even get time to let the baby suck.” *(FD1, N7)*.
  “For me I don’t have anybody to help me, so what I do is, I have to break and make sure that she get[s] all the attention that she needs.” *(FD2, N5)*.
- Get support from co-workers.
  “I asked a colleague to help in taking care of my work whiles I breastfeed”.
  “It is not easy but with time, I did adjust and colleagues also assisted me with the baby when there is pressure.” *(FD3, N4)*.
- Breastfeed in cars or offices.
  “For me I don’t have anybody to help me take care of the child during working hours, so what I do is, I have to take a break from my work and breastfeed her in the office.” *(FD1, N1)*.
  “During working hours, I breastfed my baby in my car or express milk in a feeding bottle.” *(FD2, N3)*.
- Add annual leave to maternity leave.

“For me after my maternity leave, I take my annual leave and make sure that I have time to breastfeed the child, so before I come back to work the baby is somewhere five getting to six months, so I can go ahead to get some water or small porridge to continue breastfeeding so that I can also work.” *(FD3, N5)*.

### Family and social support strategies

- Send babies to daycare.
  “Leave child at daycare and breastfeed during breaks.” *(FD2, N1)*.
  “Had to send the child to a daycare around the workplace.” *(FD3, N7)*.
- Support from family members.
  “I had to take my sister to the workplace to support me, so that I can breastfeed the child as and when the child needs.” *(FD1, N6)*.
  “I had my mother-in-law in the house who could take care of my child, so I just express and then leave it behind.” *(FD2, N5)*.
- Alternative feeding approaches.
  “I make sure that she eats a lot of the porridge or other foods that I have prepared for her and the breastmilk I use that one to support her when I am at work but when I get to the house, all the time that she needs, I have to give to her, so I am the walking food when I come to the work.” *(FD3, N7)*.
  “I had to add infant formula to supplement the breastmilk.” *(FD1, N5)*.

### Links and summary

**Fig. 2:**
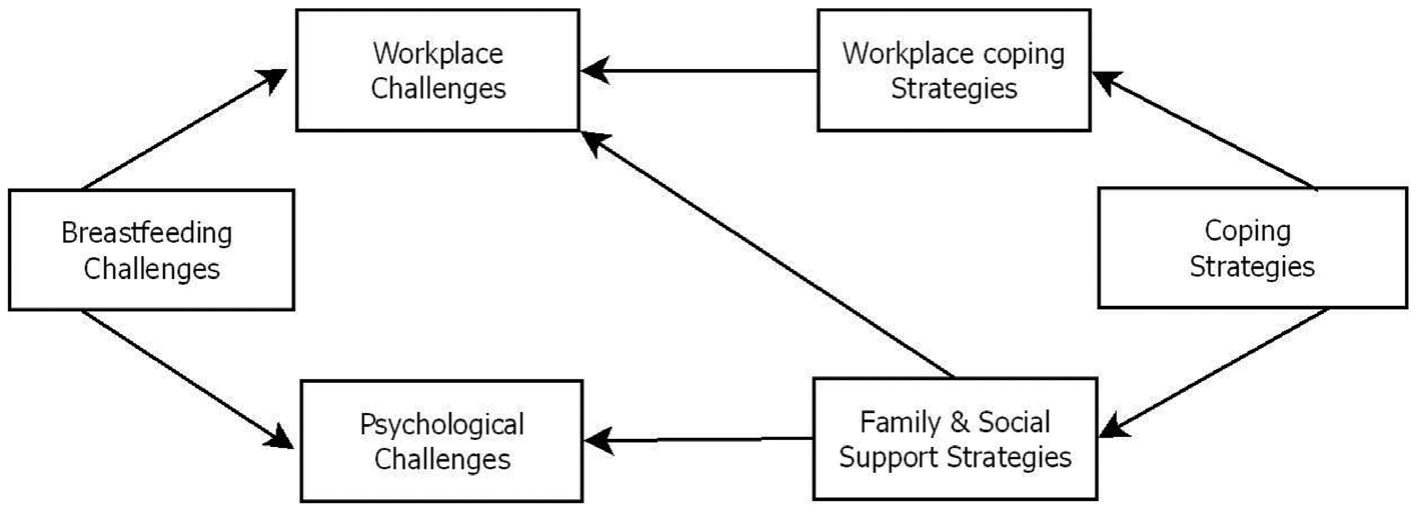
Links between workplace breastfeeding challenges and coping strategies

To sum up both qualitative and quantitative results, workplace breastfeeding challenges were divided into two categories: workplace challenges and psychological challenges (Fig. 2). To lessen workplace challenges, mothers turned to family and social support systems as well as workplace adaption techniques. Family and social support measures were largely used to manage psychological challenges (Fig. 2).

**Fig. 3:**
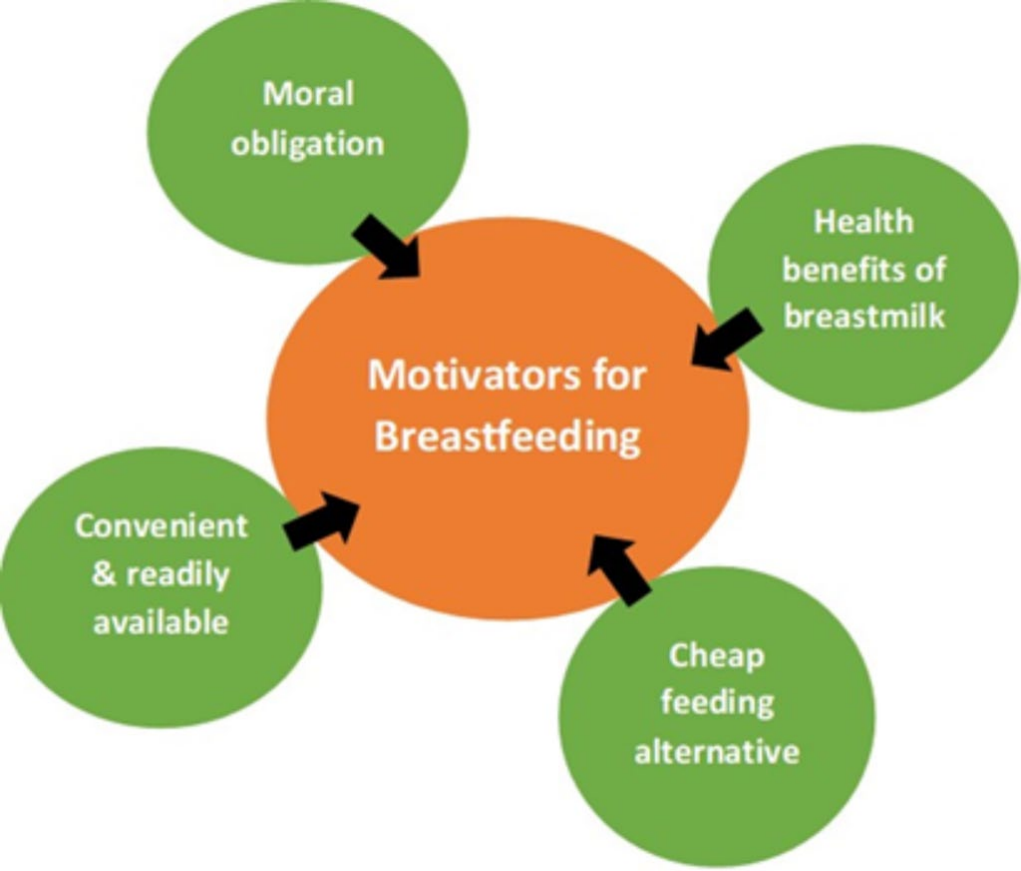
Motivators for breastfeeding among women in institutions without complete breastfeeding support environment

Figure 3 presents the motivators for continuous breastfeeding at workplaces without complete BFSE. Four key motivators were identified:

- Health benefits of breastmilk: mothers were well informed of the benefits of breastmilk for their children and they strived to breastfeed in spite of challenges encountered at the workplace. The underlisted gives some details of mothers’ expressions of their motivations on health benefits of breastmilk:
  “Breastmilk contains all the nutrients that the child needs, from birth up to six months. So if you do not breastfeed the child well with the breastmilk and you go with the formula feeds, the child will end up by getting so many infections and sickness, so, because of that one, I prefer to continue with the breastmilk alongside my work.” *(FD1, N8)*.
  “I would continue to breastfeed because of the benefits of breastmilk to the child and to myself, so that motivates me at any point to try my best to breastfeed the child.” *(FD3, N6)*.
- Moral obligation: mothers also felt that they had a moral obligation to breastfeed as breastfeeding advocates, additionally breastmilk is a gift from God for the child which must be given (moral obligation).
  We are preaching exclusive breastfeeding, so we have to show good example, if you leave the baby at home you need to express the breastmilk.
  “Breastmilk is God’s gift for every child, mothers must not deny children what rightly belongs to them.” *(FD2, N7)*.
- Cheap feeding alternative: some mothers also indicated that it was cheap to breastfeed as one did not have to buy breastmilk (cheap feeding alternative).
  “We all know that supplementary feeding is very expensive, so why not give my child breastmilk which is readily available.” *(FD2, N4)*.
  “If you breastfeed, it saves money and time.” *(FD1, N3)*.
- Convenience and readily available nature of breastmilk: other mothers indicated that breastfeeding was convenient; they did not have to cook; breastmilk is readily available at anytime and anywhere (convenience and readily available nature of breastmilk).
  “I don’t have to be thinking of what my baby will eat, no hustle at all. . ” (FD3, N1).
  “Breastmilk is ready for feeding, you don’t need to mix, boil water and all those things. So those are some of the things that motivate me to breastfeed my child.” *(FD2, N8)*.

All [39] management representatives of the health facilities were not aware that their respective facilities needed to have a specific workplace breastfeeding policy that fed into the national policy agenda. In their state of unawareness of the need to have a specific workplace breastfeeding policy, they expressed their views in diverse ways as indicated below:

“Health workers are citizens and all citizens are equal, so a policy for breastfeeding babies is a policy for everybody. So, it is not specific to health, education: no. It is breastfeeding policy for Ghana and so it is not divided into institutions.” *(ID, N18)*.

“Normally we don’t formulate our own policies. The policies that are formulated by Government is what we also use at the operational level. I think the policies are still in the making. All that we know is that the child should be breastfed for two years and beyond, exclusively for six months.” *(ID, N14)*.

## Discussion

### Breastfeeding supportive environment

Out of the 4-point checklist used in this study to assess the facilities’ BFSE status, no facility had complete BFSE. All the facilities offered flexible breaks, but all lacked a lactation support policy and back-to-work education for co-workers. Only one facility had a designated room / private space for breastfeeding. Our findings on complete BFSE are similar to an earlier study conducted in Ghana [15] which reported inadequate breastfeeding support at workplaces for staff willing to breastfeed. Similarly, our current findings are not different from what prevailed (poor BFSE) over a decade ago among hospitals in Hong Kong [16].

Overall, most participants breastfed their children at work. This makes the workplace environment a crucial contributing factor for optimal breastfeeding. Our findings suggest that the institutions tasked with the mandate to advocate for breastfeeding do very little to support their own staff to sustain optimal breastfeeding. Nearly all the facilities in this study did not have designated or private rooms for the purposes of breastmilk expression and / or breastfeeding. Possible explanations per anecdotal evidence for this finding could be due to the general belief that the health facilities were already designated as baby friendly facilities by the Ghana Health Service, unless in places (like the wards, x-ray units, etc.) where the risk of injuries or infection were said to be detrimental to the health of both baby and mother. Similarly, per anecdotal evidence, at the lower level facilities such as the CHPS compounds, the health staff reside and work in the same facilities and probably did not see the need to have designated or private rooms for breastfeeding. Similar to this current study and its finding, some other studies in Hong Kong [16], and Australia [17] indicated that less than 30% of health institutions had designated breastfeeding rooms. A recent study in Ghana indicated that less than 10% of educational and healthcare institutions [18] had lactation rooms for staff which resonates with our findings. Similarly, in other studies (though not specifically on health institutions), breastfeeding rooms are non-existent across the different sectors in Ghana [11, 19–21].

Furthermore, on institutional support for its staff to breastfeed, none of the facilities in our study provided any form of education for coworkers when breastfeeding health workers returned from maternity leave. A possible interpretation is that health staff were the main educators of health and nutrition information to the general public and such efforts were not made to give co-workers back-to-work breastfeeding education. Additionally, none of the facilities had a lactation support policy. Similar findings in studies at Michigan State reported the nonexistence of a workplace breastfeeding policy and / or support [22, 23]. On the contrary, Dinour and Szaro [24] in their systematic review found that most employers do have comprehensive breastfeeding support practices and policies.

The absence of a lactation support policy may result in early breastfeeding cessation due to an inadequate workplace BFSE [25]. The nonexistence of a workplace breastfeeding support policy might be a contributing factor to the observed declining EBF rates among professional workers [11].

Finally, on institutional support for its staff to breastfeed, all health facilities provided “flexible breaks” for their staff. Aside from the 90 days maternity leave available for all lactating mothers in Ghana, health institutions in this study provided informal and unstructured breaks for breastfeeding. Nearly all the lactating workers breastfed their infants four or more times (breastfeeding on demand) during working hours. These breastfeeding hours are considered and paid for as working hours. Despite the poor BFSE, workers are committed to breastfeeding and are able to succeed at breastfeeding in the workplace due to their knowledge of the health benefits of breastfeeding for mother and child [26–28]. ‘Flexible breaks’ is the topmost support provided to lactating staff in this study, a similar finding to a reviewed outcome of other studies [24]. Conversely, only few employees of hospitals in Hong Kong were allowed to take breastfeeding breaks [16].

### Workplace breastfeeding challenges

More women including lactating mothers are joining the workforce [29]. This has challenged the natural and biological role of women providing breastmilk at regular intervals and / or on demand to ensure their children are well nourished. Challenges differ depending on the kind of support women receive at their respective workplaces. Lack of private space for breastfeeding; inadequate work options; no support from co-workers and management; emotional stress; and inadequate breastfeeding breaks emerged as key breastfeeding challenges in this study.

The topmost challenge faced by lactating workers in the current study was the difficulty of combining work schedules with breastfeeding; almost four out of ten respondents had this challenge. Breast milk production is trigged by hormonal responses spurred by adequate and continuous stimulation from suckling [30]. This mechanism however, may not be well suited in the hospital setting, where time is of essence for the survival or treatment outcome of critical cases, coupled with heavy workload and inadequate health staff particularly in peripheral facilities [31, 32]. As such, mothers’ ability to adequately breastfeed or express may be hindered since the timing and treatment outcomes of clinical cases cannot be predicted. Similar studies [12, 33–36] indicated that work-related schedules affect breastfeeding duration among lactating workers / nurses. For BFSE to be fully realized, breastfeeding breaks together with lunch breaks should be combined for lactating staff [35] as breastfeeding breaks were said to be short. This arrangement however, may be difficult because the timing and treatment outcomes of cases cannot be predicted in the health facilities.

Another major challenge encountered by the lactating workers was the non-availability of designated breastfeeding rooms. Infections are more prevalent in hospital settings, and additionally, special units such as the radiology department possess additional risks of exposure to radiation [31, 37] making it unsafe to breastfeed in unapproved settings within health facilities. On this premise, it is understandable why the lactating staff indicated the absence of designated breastfeeding rooms as a key challenge for breastfeeding at the workplace. A recent study in Ghana indicated the unavailability of lactation rooms for health staff [18] which is similar to our findings. Similarly in other studies, breastfeeding rooms are non-existent across the different sectors in Ghana [11, 19–21]. These findings suggest the need for some specifics for the BFSE at workplaces.

Emotional stress was another challenge experienced by the lactating staff who breastfed their babies within the context of the work environment. Breastfeeding and child caring practices are stressful exercises in themselves [38, 39] which are further compounded by workload and clients demands in the health facilities. The finding from this study is similar to that of earlier studies in Australia [40] and Ghana [19] which found increased pre-return to work stress levels among mothers trying to find suitable options to breastfeed their children.

Furthermore, on breastfeeding challenges, lack of support from co-workers and management also emerged as a notable challenge encountered by the lactating workers. Our finding is similar to those of some other studies [20, 35, 41]. Additionally, inadequate work options was another challenge encountered by lactating mothers which is consonant with some other findings [35, 42, 43]. The possible explanation for our findings could be due to workload and inadequate staffing. In the study’s setting, staff are faced with heavy workloads due to inadequate staffing [44–46]. Co-workers and management may see lactating mothers as already having enough support: in addition to breastfeeding breaks, lactating mothers are often given two hours’ concession of being permitted to report to work an hour late and finish an hour early.

### Workplace breastfeeding coping mechanisms

Breastfeeding is a cultural norm in Ghana and there is an inherent and indirect social pressure to breastfeed. With workplaces challenges, the natural and biological role of women providing breastmilk at regular intervals and / or on demand becomes a daunting task. The majority of the lactating workers adapted to the challenges of inadequate breastfeeding breaks and inadequate work options by obtaining help from caretakers (mothers-in-law and older siblings) who went to the workplace together with the staff. Our finding is similar to some earlier studies which found family members’ support to be a pivotal breastfeeding coping strategy [34, 40, 47, 48]. Aside from family support, some mothers had to carry their babies on their back while working. This action could expose the children to infections depending on the assigned wards or work setting of the mothers. Few mothers send their babies to daycare centers and others leave children at home under the care of relatives.

On the challenge of lack of private space for breastfeeding, staff had to improvise and rally support to meet this need. In our study, some staff resorted to breastfeeding in cars. In some institutions, staff used the facility manager’s office for breastfeeding. For example, a manager explained that:

“Per all our health facilities and district health directorate, actually space is a challenge, we don’t have any special space for breastfeeding but we identified the in-charge’s office because at that place he or she might not be seeing clients all the time, to breastfeed our babies adequately”.

Our finding is similar to an Australian study, in which, lactating mothers on a university campus had to rally support to set up a family room to enable them to breastfeed their children [40]. Additionally, employees in Pennsylvania, USA also resorted to the use of offices for breastfeeding [2], other lactating mothers used non-breastfeeding designated rooms in Spain [49] while still others used lobbies, common-rooms for juniors or under trees for breastfeeding in Ghana [19].

For the challenge of no support from co-workers and management, lactating staff had to actively identify colleagues who were less engaged and willing to provide support services for their children. In some instances, lactating staff requested to be reassigned to different schedules or duties to enable them to breastfeed, which did create tension sometimes between staff. Our findings of reassignment is similar to job sharing as a coping mechanism for breastfeeding in work environment as reported by Payton et al., [2].

Some participants, realizing the difficulty of working and breastfeeding at the same time, adapted a coping strategy of extending their three-months mandatory maternity leave to four months (120 work days) by requesting annual leave which is an entitlement for all workers. This arrangement helped prolong the breastfeeding duration of some mothers who otherwise would have stopped after the three months’ maternity leave. Maternity leave extension was observed in other studies as a coping mechanism employed by lactating staff to enable them keep their jobs while breastfeeding their children [2]. The majority of the lactating mothers adapted to emotional stress by obtaining help from caretakers (mothers-in-law and older siblings) who stay at home to take care of their children. These caregivers were responsible for feeding and caring for the babies while the mother was at work. Our finding is similar to some earlier studies which found family members’ support to be a pivotal breastfeeding coping strategy [34, 40, 47, 48]. Few mothers send their babies to daycare centers to reduce the stress of working and feeding the child at the same time.

### Motivators for breastfeeding at the workplace

Breastmilk is the best natural infant food available to almost all mothers after delivery. This study explores the factors that motivated the breastfeeding mothers in health institutions to continue to breastfeed amid the challenges that they encountered. Health benefits of breastmilk; moral obligation; cheap feeding alternative; and convenience and readily available nature of breastmilk emerged as key breastfeeding motivators in this study.

The majority of the lactating workers were motivated to breastfeed because of the enormous health benefits of the breast milk for mother and child. This study’s finding on health benefits of breast milk is consistent with other studies [26–28]. Similarly, other studies reported that staff beliefs about the benefits of breastfeeding in the prevention of childhood illnesses, boosting of immunity, and facilitating brain development increased mothers’ commitment and determination to breastfeed [27, 50, 51].

Another motivation for the mothers is that they are morally obligated to breastfeed their infants. For these mothers, breastmilk is the God-given food meant for all infants and mothers are obliged to give such food to their babies. Morality and moral obligations are largely enshrined in cultural norms and religious beliefs. Additionally, mothers are obliged to breastfeed as they see themselves as the advocates for breastfeeding.

Some mothers also indicated that breastmilk, being a cheap feeding alternative, motivated them to continue to breastfeed. Breastmilk is undoubtedly a cheaper option for feeding infants since one does not have to buy it. This finding is in agreement with other studies [27, 50]. Additionally, it takes away the time cost of how and where mothers can access different food sources for their infants, while keeping to their jobs [19, 40].

Finally, mothers indicated that the convenience and readily available nature of breastmilk motivated them to continue to breastfeed. The convenience and readily available nature of breastmilk takes away the burden and stress of how and where mothers can access different food sources for their infants, while keeping to their jobs [19, 40]. Our finding, in a low socio-economic setting, buttresses the United Nations Children’s Fund’s assertion that mothers of low socio-economic background find breastfeeding a convenient feeding alternative [50].

### Management awareness level on workplace breastfeeding support policy

Employers stand to benefit a lot when a workplace support environment for breastfeeding is provided [52]. Additionally, enabling BFSE improves child health outcomes and reduces lactating mothers’ absenteeism due to fewer illnesses among their children [53]. Underscoring the importance of breastfeeding, the International Labour Organization highlighted the importance of maternity protection which include workplace BFSE [35]. Unfortunately, most countries still lag behind in providing workplace breastfeeding support policies due to non-awareness or unwillingness to create them.

In our study, all the management representatives of the various health facilities did not have and were not aware that their respective facilities needed to have a specific workplace breastfeeding policy that fed into the national policy agenda. Our findings are similar to studies in Michigan, Pennsylvania, and New Jersey which indicated non-awareness or absence of a workplace breastfeeding support policy among most employers [2, 22, 54]. Other studies indicated that most national organizations did not have workplace breastfeeding policies to enable the creation of a workplace BFSE [55].

The non-awareness levels of the need for specific a workplace breastfeeding support policy among the custodians of breastfeeding in our study setting is unacceptable and needs to be addressed. Furthermore, contrary to the assertion that health facilities do not have to formulate their own BFSE policies, the WHO charged all health facilities to own, display and be able to explain some elements of their policies [13, 31]. Additionally, to a large extent, this finding suggests that managers whose duty is to provide and ensure the smooth formulation and implementation of a lactation support policy do not themselves see the need to ensure a BFSE for their staff; this could be borne out of ignorance among other reasons. This assertion is evidenced by some responses from managers:

“Health workers are citizens and all citizens are equal, so a policy for breastfeeding babies, is a policy for everybody. So, it is not specific to health, education, no. it is breastfeeding policy for Ghana and so it is not divided into institutions”.

To address the non-awareness levels of the need for a specific workplace breastfeeding support policy, the regional health directorate and management representatives of all health facilities in the Upper East Region could be given an orientation on the BFSE.

This study is not without limitations and strengths. The strengths of this study include the use of both quantitative and qualitative methodological approaches. To the best of the authors’ knowledge this is the first study to specifically investigate the workplace BFSE, challenges, motivators, and coping strategies of women in health institutions in the research setting. The study may serve as the basis for further national level studies on the workplace BFSE in Ghana. We acknowledge some limitations in this study. Social desirability bias might have influenced data from the semi-structured interview as information obtained was self-reported. Furthermore, the study could not establish a cause-effect relationship since it was a cross-sectional survey.

## Conclusion

All health facilities had an incomplete BFSE. Management staff of health facilities did not have and were not aware that their respective facilities needed to have a specific workplace breastfeeding policy that fed into the national policy agenda. Our findings suggest that health workers have poor BFSE and are faced with numerous breastfeeding challenges. These challenges can be categorized as: workplace challenges and psychological challenges. To cope with workplace challenges, mothers turned to family and social support systems as well as workplace adaption techniques. Family and social support measures were largely used to manage psychological challenges. There is a need for specialized initiatives led by the regional health directorate to enhance BFSE in healthcare institutions. All health facilities in the Upper East Region should be given the responsibility of setting up breastfeeding areas for breastfeeding staff within their facilities. Management representatives from all health facilities should also attend a BFSE orientation workshop, which will help them create facility-based breastfeeding policies and guidelines for their individual institutions. Future studies are required to evaluate the effect of BFSE on work productivity, EBF, and breastfeeding duration in the health facilities.

## Data Availability

The datasets used during the current study are available from the corresponding author on reasonable request.

## List of Abbreviations

BFSE: Breastfeeding support environment
CHPS: Community-based Health Planning and Services;
EBF: Exclusive breastfeeding
WHO: World Health Organization
